# Reported health, social support, stress and associations with choline intake in pregnant women in central South Africa: the NuEMI study 2018–2019

**DOI:** 10.1186/s13690-023-01061-y

**Published:** 2023-03-30

**Authors:** Liska Robb, Elizabeth Margaretha Jordaan, Gina Joubert, Jennifer Ngounda, Corinna May Walsh

**Affiliations:** 1 Department of Nutrition and Dietetics, School of Health and Rehabilitation Sciences, Faculty of Health Sciences, University of the Free State, PO Box 339, internal box G24, 9300 Bloemfontein, Republic of South Africa; 2 Department of Biostatistics, School of Biomedical Sciences, Faculty of Health Sciences, University of the Free State, Bloemfontein, Republic of South Africa

**Keywords:** Maternal nutrition, Pregnancy, Health, Social support, Stress, Choline, South Africa, NuEMI

## Abstract

**Background:**

The health and well-being of pregnant women can influence pregnancy outcomes and are closely associated with social support and experiences of stress. Poor nutrition predisposes to poor health with choline intake affecting pregnancy outcome. This study determined reported health, social support, and stress and how these factors are associated with choline intake in pregnancy.

**Methods:**

A cross sectional study was performed. Pregnant women in their second and third trimesters attending a high-risk antenatal clinic at a regional hospital in Bloemfontein, South Africa, were included. Trained fieldworkers obtained information during structured interviews using standardised questionnaires. Logistic regression with backward selection (*p* < 0.05) was used to select significant independent factors associated with choline intake. Variables with a *p*-value < 0.15 in bivariate analysis were considered for inclusion in the model.

**Results:**

Median age and gestation in the sample (N = 682) were 31.8 years and 32.0 weeks, respectively. Most participants (84.7%) consumed less than the adequate intake (AI) of 450 mg of choline per day. Most participants (69.0%) were either overweight or obese. One in eight participants (12.6%) reported not having anyone that could help them in times of need, more than one third (36.0%) reported having unpayable debt and one in twelve (8.4%) reported experiencing physical abuse by their partners. Normotensive participants and those using anti-retroviral therapy (ART) (thus HIV-infected), were more likely to consume choline in amounts below the AI (*p* = 0.042 and *p* = 0.011, respectively). Logistic regression analysis showed that the odds of consuming choline in amounts below the AI were lower for participants that were not using ART versus those using ART, with an odds ratio of 0.53.

**Conclusion:**

HIV-infected participants were more likely to consume choline in levels below the AI. This vulnerable group should be the focus of targeted efforts to improve choline intake.

## Background

Maternal and foetal health outcomes are significantly affected by nutritional status during pregnancy and inadequate intake of essential nutrients during pregnancy impacts negatively on maternal and child health in the short- and long-term [1]. Rapid and significant developmental and physiological changes occur during this period and to support these changes, nutritional requirements increase [2]. Several nutrients, including the micronutrient choline, are especially important to support optimal short- and long-term pregnancy outcomes. Choline is a methyl donor involved in one-carbon metabolism affecting DNA and histone methylation – processes that ultimately regulate gene expression [3]. Choline is also involved in normal placental functioning, as a high choline intake is important for placental angiogenesis [4]. There is a higher need for choline during pregnancy as this nutrient is also vital for normal foetal brain development [5]. Zeisel (2017) suggests that our understanding of developmental abnormalities and the origins of chronic diseases can be better informed by appreciating the influence of methyl-donor nutrients, such as choline, on epigenetic programming [6].

Non-communicable diseases (NCDs) are responsible for the majority of deaths in the world. The primary NCDs, including cardiovascular and lung diseases, cancers and diabetes, account for 38 of the 41 million NCD-related deaths every year. The World Health Organization (WHO) focuses on preventing NCD-related deaths by attempting to reduce the major risk factors which include unhealthy diets, tobacco use, the harmful use of alcohol and physical inactivity [7]. In South Africa, NCDs are highly prevalent and a major cause for concern. The 2016 South African Demographic and Health Survey (SADHS) (participants > 15 years) revealed that 68.0% of women were overweight or obese, while the prevalence of hypertension and diabetes in women was 46.0% and 13.0% respectively [8]. In addition to the challenge of NCDs in South Africa, prevalence rates of human immunodeficiency virus (HIV) remain high, especially in women of reproductive age. Prevalence of HIV in women between the ages of 25 and 44 years ranges from 36.0 to 40.3% [9].

In order to contribute to the health of the mother and infant, antenatal care (ANC) is vital as it provides an opportunity to monitor and screen pregnant women and to identify health risks at an early stage when interventions can positively impact on pregnancy outcome. According to the most recent SADHS, most women in South Africa (94.0%) received ANC from a skilled provider during their most recent pregnancy. Almost all of these women had their blood pressure measured, provided a blood and urine sample and were advised about alcohol and tobacco use [9]. In addition to the mentioned variables, maternal psychological and social stresses are important health-related aspects that must not be neglected during pregnancy. There is evidence to suggest that maternal stress during pregnancy can have major epigenetic effects, contributing to adverse physical and mental effects in the child [10].

As stated by Kuddus and Rahman (2016), health and nutrition cannot entirely be separated. Poor health can cause poor nutrition, as intake and use of nutritious foods and nutrients may be decreased, while poor nutrition can contribute to poor health [11]. Adequate intake of choline during pregnancy contributes to overall good nutrition and as such may contribute to good health of the mother and optimal development of the foetus. Thus, the main aim of the study was to determine reported health, social support and stress and associations with choline intake of pregnant women that participated in the Nutritional status of Expectant Mothers and their newborn Infants (NuEMI) study.

## Methodology

A cross-sectional study was undertaken. All pregnant women attending the high-risk antenatal clinic at Pelonomi Hospital, Bloemfontein, South Africa from May 2018 to April 2019 were eligible to participate in this study. This is a high-risk clinic to which older women (> 35 years), and women with multiple pregnancies, previous poor pregnancy outcomes (neonatal death and preterm delivery), two or more previous caesarean sections, a gravida of six or more, obesity, hypertension and/or diabetes mellitus are referred from surrounding areas and towns. A consecutive convenience sample of 682 pregnant women in their second and third trimesters was included.

### Dietary intake

Information about dietary intake (N = 681) was determined using a quantitative food frequency questionnaire (QFFQ). Choline intake was determined using data obtained from the QFFQ. The South African Food Composition Database (SA-FCDB) does not contain data on choline content of foods; thus, all food items were first matched to foods in the USDA Database for the Choline Content of Common Foods (Release 2) [12] using the FAO/INFOODS Guidelines for Food Matching [13]. Choline intake was compared to the 1998 dietary reference intake (DRI) values set by the Institute of Medicine (IOM) of the United States of America (USA), currently known as the National Academy of Medicine (NAM) [14]. Although the estimated average requirement (EAR) should be used to evaluate population intakes of nutrients, no EAR values are yet available for choline and therefore the AI value for pregnant women (19–70 years) of 450 mg/day and the tolerable upper intake level (UL) for all adults of 3500 mg/day [14] were used in this study.

### Reported health, social support and stress

Information related to social support (group membership, network of friends, family structure), tobacco and alcohol use, medical and pregnancy history, medications, presence of stress and behaviours related to the control of stress were obtained using the Reported Health and Lifestyle Questionnaire. This questionnaire was adapted from the Antenatal Questionnaire of the Birth to Twenty (BTT) study [15], a longitudinal study focussed on child and adolescent health and development in Africa.

### Gestational body mass index (GBMI)

Current weight and height of each participant were measured and entered into an algorithm of Davies et al. (2013) along with gestation in weeks to calculate GBMI. Gestational body mass index was categorised as follows: ≥ 10 to < 19.8 kg/m^2^ (underweight), ≥ 19.8 to < 26.1 kg/m^2^ (normal weight), ≥ 26.1 to < 29 kg/m^2^ (overweight), and ≥ 29 to < 50 kg/m^2^ (obese) [16]. Participants with a GBMI ≥ 50 kg/m^2^) were included in the “obese” category.

### Validity and reliability

The QFFQ used to determine nutrient intake was adapted from a QFFQ previously validated for use in South Africa [17–19]. Researchers and fieldworkers received comprehensive training on dietary intake assessment methodology. The use of dietary intake assessment kits specifically developed for the current study increased reliability, as all fieldworkers used comparable measures for such items as different sized spoons, cups, plates, and bean bags with known volumes to obtain the dietary intake information. Household food measures that were available in local shops were included in the kits. All dietary intake data were checked and coded by only two researchers, both registered dietitians.

Equipment used to obtain anthropometric data were calibrated and the measurements were taken using standardised, established techniques as described in the literature [20]. To further ensure reliability, each measurement was taken three times and the mean value used in analysis. The scale was calibrated with a known weight after every 20th participant was weighed.

Interviews were conducted in the participant’s preferred language (English, Afrikaans, or Sesotho) to ensure that participants understood what was being asked and provided reliable answers.

### Data analysis

Collected data were entered into Excel documents. Data cleaning and statistical analysis were performed by the Department of Biostatistics, Faculty of Health Sciences, University of the Free State. Data were analysed using SAS/STAT software, Version 9.4 of the SAS system for Windows, Copyright © 2013 SAS Institute Inc. SAS and all other SAS Institute Inc. product or service names are registered trademarks or trademarks of SAS Institute Inc., Cary, NC, USA [21].

Descriptive statistics, including frequencies and percentages for categorical data, and means and standard deviations (SDs) for symmetrical numerical variables, or medians and interquartile range (IQR) for skew numerical variables were calculated. Due to occasional missing values (for example one participant did not have dietary information) and subgroup analyses, the number of cases with information are indicated throughout. Differences between groups were assessed by *p*-values (t-tests for symmetrical numerical variables, Mann-Whitney tests for skew numerical variables, chi-squared tests for categorical variables or Fisher’s exact tests for categorical variables with sparse data) or 95% confidence intervals (CIs) for median, mean or percentage differences. Logistic regression with backward selection (*p* < 0.05) was used to select significant independent factors associated with choline intake. Variables with a *p*-value of < 0.15 in bivariate analysis were considered for inclusion in the model.

### Ethical considerations

Ethics approval was obtained from the Health Sciences Research Ethics Committee of the University of the Free State (UFS-HSD2018/0625/2603) and the Free State Department of Health. Participants who provided written informed consent were included. To ensure confidentiality, each participant was allocated a unique number and no information that could identify individuals was used in data analysis.

## Results

The median age of the sample (N = 682) was 31.8 years (IQR 26.8–36.5 years). Median gestation was 32.0 weeks (IQR 26–36 weeks). Most participants were pregnant with one baby (93.4%), while 6.6% expected twins.

### Choline intake

Median daily choline intake (N = 681) was 275.0 mg (IQR: 84.7 mg – 386.7 mg). Most participants consumed less than the adequate intake (AI) of 450 mg/day for choline (84.7%), while 15.3% consumed a value between the AI and the UL (3500 mg/day) [14].

### Alcohol and tobacco use

Sample prevalence of alcohol use and smoking and associations thereof with choline intake are presented in Table 1. Considering all participants, 6.2% smoked, and 9.0% consumed alcohol during the current pregnancy. Most of the current smokers did so daily (35/42; 83.3%). Almost one in ten participants (8.8%) snuffed or chewed tobacco during pregnancy. Smoking and alcohol consumption were not significantly associated with choline intake in this sample.

**Table 1:** Sample prevalence of alcohol use and smoking and associations thereof with choline intake in pregnant women in central South Africa: the NuEMI study 2018–2019

	Sample prevalence (N = 680)		Choline intake < 450 mg		Choline intake ≥ 450 mg		*p*-value
	n	%	n	%	n	%	
**Current cigarette smoker**							0.799
Yes	42	6.2	35	83.3	7	16.7	
No	638	93.8	541	84.8	97	15.2	
**Current alcohol user**							0.902
Yes	61	9.0	52	85.3	9	14.8	
No	619	91.0	524	84.7	95	15.4	
**Combined use of cigarettes and alcohol**							0.728
Both	14	2.1	11	78.57	3	21.4	
Either	75	11.0	65	86.7	10	13.3	
Neither	591	86.9	500	84.6	91	15.4	

*p*-value for Chi-square or Fisher’s exact test for categorical data with significance set at *p* < 0.05.

### Pregnancy health

Most participants (89.6%) had been pregnant before. Almost a quarter of participants were hospitalised at some stage during the current pregnancy (23.5%), with the main reported reasons for hospitalisation being abdominal pain (26.6%), hypertension (18.5%) and vaginal bleeding (5.2%). Symptoms experienced during the current pregnancy included loss of appetite (60.8%), nausea (56.6%), vomiting (54.8%), swelling of the feet (49.6%) and constipation (39.2%). Several participants experienced weight loss of more than 3 kg (17.2%), and diarrhoea for more than three days (13.8%).

Self-reported disease diagnoses of participants are summarised in Table 2. Hypertension (23.0%) and sexually transmitted diseases (STDs) (18.5%) were the main conditions that had been diagnosed.

**Table 2:** Self-reported diagnoses of pregnant women in central South Africa: the NuEMI study 2018–2019

	Currently		Previously		Never	
	n	%	n	%	n	%
Hypertension (N = 682)	157	23.0	95	13.9	430	63.1
Heart disease (N = 681)	4	0.6	17	2.5	660	96.9
Diabetes mellitus (N = 681)	31	4.6	7	1.0	643	94.4
Tuberculosis (TB) (N = 681)	7	1.0	29	4.5	645	94.7
Asthma (N = 681)	21	3.1	26	3.8	634	93.1
Any sexually transmitted disease (STD) (N = 682)	126	18.5	6	10.1	487	71.4
Vaginal infection/discharge (N = 682)	106	15.4	91	13.3	485	71.1
Cancer (N = 681)	0	0	4	0.6	677	99.4
Lung diseases (N = 681)	1	0.2	6	0.9	674	99.0
Elevated cholesterol (N = 680)	3	0.4	7	1.0	670	98.5
Stroke (N = 680)	1	0.2	10	1.5	669	98.4

A relatively large percentage of participants used medication for hypertension management (23.7%) and antiretroviral therapy (ART) (31.8%), while 3.7% used oral glucose lowering medication.

Table 3 summarises associations between reported health related to pregnancy and choline intake. Participants who did not have hypertension and those who were using ART (thus HIV-infected) had significantly lower choline intakes.

**Table 3:** Associations between reported health related to pregnancy and choline intake in pregnant women in central South Africa: the NuEMI study 2018–2019

	n	Choline intake < 450 mg		Choline intake ≥ 450 mg		*p*-value
		n	%	n	%	
**Previous pregnancy**						0.091
Yes	610	512	83.9	98	16.1	
No	71	65	91.6	6	8.5	
**Hospitalisation during pregnancy**						0.887
Yes	160	135	84.8	25	15.6	
No	123	442	84.8	79	15.2	
**Diarrhoea lasting > three days**						0.414
Yes	94	77	81.9	17	18.1	
No	587	500	85.2	87	14.8	
**Constipation**						0.357
Yes	267	222	83.2	45	16.9	
No	414	355	85.8	59	14.3	
**Nausea**						0.278
Yes	386	322	83.4	64	16.6	
No	295	255	86.4	40	13.6	
**Vomiting**						0.378
Yes	374	321	85.8	53	14.2	
No	307	256	83.4	51	16.6	
**Appetite loss**						0.903
Yes	413	351	85.0	62	15.0	
No	267	226	84.6	41	15.4	
**Weight loss > 3 kg**						0.165
Yes	117	104	89.0	13	11.1	
No	562	471	83.8	91	16.2	
**Heartburn**						0.306
Yes	90	73	81.1	17	18.9	
No	591	504	85.3	87	14.7	
**Hypertension**						*0.042
Yes	157	125	79.6	32	20.4	
No	524	452	86.3	72	13.7	
**Diabetes**						0.302
Yes	31	24	77.4	7	22.6	
No	649	552	85.1	97	15.0	
**Any sexually transmitted disease**						0.051
Yes	125	113	90.4	12	9.6	
No	556	464	83.5	92	16.6	
**Tuberculosis**						0.603
Yes	7	7	100.0	0	0.0	
No	673	569	84.6	104	15.5	
**Use of antiretroviral therapy**						*0.011
Yes	216	194	89.8	22	10.2	
No	463	381	82.3	82	17.7	
**Use of Tuberculosis medication**						0.228
Yes	21	20	95.2	1	4.8	
No	658	555	84.4	103	15.7	
**Use of glucose-lowering medication**						1.000
Yes	251	21	84.0	4	16.0	
No	654	554	84.7	100	15.3	

*p*-value for Chi-square or Fisher’s exact test for categorical data with significance set at *p* < 0.05; *indicates significance.

### Gestational body mass index

Gestational body mass index classification and associations between GBMI of participants and choline intake are presented in Table 4. Gestational body mass index was only calculated for participants who were pregnant with one baby, as the algorithm was not developed for multiple gestations. Most participants (69.0%) were either overweight or obese. Median GBMI (N = 637) was 30.8 kg/m^2^ (obese) with an interquartile range of 24.7 kg/m^2^ – 37.2 kg/m^2^. Although not significant, there was a trend suggesting that obese participants were less likely to consume choline in amounts below the AI than participants in the other GBMI categories.

**Table 4:** Gestational body mass index and choline intake in pregnant women in central South Africa: the NuEMI study 2018–2019

	Sample prevalence (N = 636)		Choline intake < 450 mg		Choline intake ≥ 450 mg		*p*-value
	n	%	n	%	n	%	
**Gestational body mass index**							0.086
Underweight (≥ 10 to < 19.8 kg/m^2^)	42	6.6	36	85.7	6	14.3	
Normal weight (≥ 19.8 to < 26.1 kg/m^2^)	155	24.4	139	89.7	16	10.3	
Overweight (≥ 26.1 to < 29 kg/m^2^)	77	12.1	68	88.3	9	11.7	
Obese (≥ 29 kg/m^2^)	362	56.9	295	81.5	67	18.5	

*p*-value for Chi-square or Fisher’s exact test for categorical data with significance set at *p* < 0.05.

### Social support and stress

Data related to social support and stress are presented in Table 5. One in eight participants (12.6%) did not have anybody that could help them in times of need. More than one third of participants (36.0%) experienced recent unpayable debt. A concerning finding was that one in every twelve participants experienced physical abuse at the hands of their partners (8.4%). No significant associations were found between social support and stress, and choline intake.

**Table 5:** Sample prevalence of social support and stress as well as associations thereof with choline intake in pregnant women in central South Africa: the NuEMI study 2018–2019

	Sample prevalence		Choline intake < 450 mg		Choline intake ≥ 450 mg		*p*-value
	n	%	n	%	n	%	
**Availability of people who could help the participant if a significant problem arose (N = 681)**							0.584
Nobody	86	12.6	76	88.4	10	11.6	
Unsure	35	5.1	30	85.7	5	14.3	
Multiple people	560	82.2	471	84.1	89	15.9	
**Has a husband or partner with whom she can talk to about any problem she might have (N = 665)**							0.293
Never	44	6.6	35	79.6	9	20.5	
Sometimes	150	22.6	132	88.0	18	12.0	
Always	471	70.8	394	83.7	77	16.4	
**Belongs to a church or religious organisation (N = 681)**							0.918
Yes	508	74.6	430	84.7	78	15.4	
No	173	25.4	147	85.0	26	15.0	
**Has been in danger of being killed by criminals during the past six months (N = 681)**							0.850
Yes	43	6.3	36	83.7	7	16.3	
No	638	93.7	541	84.8	97	15.2	
**During the past six months, participant witnessed a violent crime (e.g., murder, robbery, assault, rape) (N = 679)**							0.335
Yes	79	11.6	64	81.0	15	19.0	
No	600	88.6	511	85.2	89	14.8	
**Unpayable debt during the previous six months (N = 679)**							0.321
Yes	245	36.0	203	82.9	42	17.1	
No	434	63.9	372	85.7	62	14.3	
**Participant or close family members had not been able to find a job for more than six months (N = 680)**							0.524
Yes	482	70.9	411	85.3	71	14.7	
No	198	29.1	165	83.3	33	16.7	
**During the last six months, participant or anyone in her close family was seriously ill (N = 681)**							0.442
Yes	272	39.9	234	86.0	38	14.0	
No	409	60.1	343	83.9	66	16.1	
**During the last six months, any member of participant’s close family died (N = 681)**							0.407
Yes	200	29.4	173	86.5	27	13.5	
No	481	70.6	404	84.0	77	16.0	
**Someone in participant’s close family has a problem with drugs or alcohol (N = 679)**							0.315
Yes	213	31.4	176	82.6	37	17.4	
No	466	68.6	399	85.6	67	14.8	
**During the last six months, participant had a break-up with her partner (N = 676)**							0.893
Yes	107	15.8	91	85.1	16	15.0	
No	569	84.2	481	85.5	88	15.5	
**During the last six months, participant’s partner hit or beat her (N = 669)**							0.884
Yes	56	8.4	47	83.9	9	16.1	
No	613	91.6	519	84.7	94	15.3	

*p*-value for Chi-square or Fisher’s exact test for categorical data with significance set at *p* < 0.05.

### Reported health, social support and stress factors associated with inadequate choline intake: logistic regression

The following reported health, social support and stress variables were considered for inclusion in a logistic regression analysis: pregnant before (no versus yes), current hypertension (no versus yes), current sexually transmitted disease (no versus yes), current ART use (no versus yes) and GBMI (≥ 10 to < 19.8 kg/m^2^, ≥ 19.8 to < 26.1 kg/m^2^, ≥ 26.1 to < 29 kg/m^2^, ≥ 29 kg/m^2^).

Current ART use was selected in the model with an odds ratio as indicated in Table 6. The odds of having a choline intake below the AI were lower for participants that were not using ART compared to those using ART (HIV-infected) with an odds ratio of 0.53.

**Table 6:** Reported health, social support and stress factors associated with inadequate choline intake in pregnant women in central South Africa: the NuEMI study 2018–2019: logistic regression

Variable	Description	Odds ratio (95% CI)	*p*-value
Current antiretroviral therapy use	no vs. yes	0.53 (0.32; 0.87)	0.011

## Discussion

Median daily choline intake was 275.0 mg which is considerably lower than the AI of 450 mg. Adequate nutrient intake is important to support the health of the mother and baby and promotes optimal development *in utero* and during the early childhood years. For example, there is evidence that choline is required for placental health, and an optimally functioning placenta decreases the risk of preeclampsia and poor foetal growth [22]. A higher maternal choline intake can also affect gene expression, causing changes that regulate placental vascularisation, angiogenesis and stress reactivity, that may reduce stress-related disease risk in the offspring in adult life [22].

### Reported health and lifestyle

Alcohol and tobacco are well-known teratogens and can cause an array of poor health outcomes. South Africa has the highest rate of foetal alcohol spectrum disorder (FASD) in the world [23], and almost one in ten participants (9.0%) in the current study consumed alcohol during the current pregnancy. Ideally, pregnant women who consume alcohol should consume higher amounts of choline, because it has been shown that higher choline intake may protect against some of the adverse consequences of FASD [24, 25]. However, no significant associations were found between alcohol use and/ smoking cigarettes and choline intake in the current study.

Almost one quarter of participants were hospitalised during the current pregnancy, mainly due to abdominal pain, hypertension, vaginal bleeding, and vomiting. Furthermore, about one quarter of participants reported hypertension as a current diagnosis. Hypertensive participants were less likely to consume choline in amounts below the AI. Participants with a higher choline intake could have had an overall higher food (and choline) intake, which may have contributed to overweight or obesity – known risk factors for hypertension [26]. An animal study by Liu et al. (2017) found that choline ameliorated cardiovascular damage in hypertensive rats. The authors proposed that the mechanisms by which this occurred could be related to improved vagal activity and inhibition of the inflammatory response. One of the positive cardiovascular outcomes of choline supplementation was a decreased systolic blood pressure and the authors suggest choline as a possible adjunct therapy for hypertension [27]. Although it has been shown that the consumption of choline may increase cardiovascular (CVD) risk through the production of trimethylamine N-oxide (TMAO) [22], a recent meta-analysis by Meyer and Shea (2017) found no association between choline intake and CVD [28]. More research is therefore needed to determine the overall association between choline intake and CVD.

Approximately a third of participants reported using ART at the time of the study. This finding is in line with national statistics, as 25.0% of South African women of reproductive age were HIV-infected in 2019 [29]. A statistically significant association was found between the use of ART and choline intake. The odds of having a choline intake below the AI were lower for participants that were not using ART compared to those that were using ART (HIV-infected) with an odds ratio of 0.53. Various factors can contribute to a poor nutrient intake and a compromised nutritional status in HIV-infected individuals, who are more likely to eat less due to food insecurity, complications of HIV infection, or side effects of medication [30]. A study performed in rural communities in the Eastern Cape, South Africa, demonstrated that individuals from HIV-afflicted households (N = 68) consumed fewer kilojoules and had lower dietary diversity than non-HIV-afflicted households [31]. This highlights the possible effect that HIV might have on food intake causing a lower choline intake. Additionally, common symptoms occurring in pregnancy, such as nausea, constipation and appetite loss, can exacerbate a poor nutrient intake in HIV-infected women.

### Gestational body mass index

Although the majority of participants were overweight or obese, 6.6% were underweight. Both overweight [32] and underweight [33] during pregnancy are associated with a higher risk for adverse effects and poor birth outcomes. Specifically, maternal obesity may increase levels of adiposity in the foetus which may contribute to poorer metabolic health in the offspring. Jack-Roberts et al. (2017) investigated the effect of choline supplementation on lipid metabolism in pregnant mouse dams. It was shown that supplementation of choline in obese dams improved indices of foetal adiposity, probably due to changes in the expression of specific genes. The authors recommend further studies to elucidate all related mechanisms by which choline might affect foetal adiposity during periods of maternal high-fat feeding and obesity [34]. Although a statistically significant difference was not observed regarding GBMI and choline intake in the current study, there was a trend indicating that obese participants had higher choline intakes than other GBMI categories. It is likely that obese participants consumed a larger volume of food, and subsequently more choline, than others.

### Stress and social support

Situations that can cause psychological stress were common in the participants. For example, approximately one in ten participants recently witnessed a violent crime or experienced physical abuse from their partner. Joblessness, death and unpayable debt in the household or family were also commonly reported. However, most participants did have a support system in place in the form of individuals they could ask for assistance or through their membership of a church or contacts with a social organisation. A large body of evidence suggests that maternal stress can have a profound influence on birth outcomes [35–37]. Women who experienced high levels of psychological and social stresses during pregnancy have been shown to be at higher risk of preterm delivery [38–40]. Prenatal stress exposure may also adversely affect short- and long-term foetal neurodevelopment. Hormonal and immune stress mediators play an important role in normal brain development and inappropriate levels of these mediators can negatively affect brain development [41]. Moreno Gudiño et al. (2017) demonstrated that choline supplementation in male rat pups attenuated the memory dysfunction provoked by stress caused by maternal separation during important developmental periods (post-natal days 1 to 14 and days 21 to 60). The authors propose the possibility of supplementing choline to stressed adolescents to mitigate the cognitive damage caused by stress in early life [42]. Conversely, van Lee et al. (2017) found that higher maternal plasma choline levels were associated with more symptoms related to anxiety and depression during pregnancy, but no association was found between maternal plasma choline and postnatal mental well-being [43]. More research is needed regarding the possible interactions between stress, choline intake, and foetal neurodevelopment.

### Limitations

All data (except weight and height measurements) were self-reported. Convenient sampling was used instead of random sampling. The antenatal clinic where data were collected is considered a high-risk clinic, thus results cannot be generalised to low-risk pregnancies. However, participants in the current study reside in the same communities as other women with low-risk pregnancies who may be subjected to similar levels of stress and access to social support.

## Conclusions and recommendations

Few participants consumed choline in amounts above the AI, which is a concerning finding, as the importance of choline during pregnancy is well established. The AI level can generally not be used to determine prevalence of deficiency in a population. However, if the intake is above the AI, prevalence of inadequate nutrient intakes is likely to be low. Alcohol use in this sample was relatively high. As choline supplementation has been shown to mitigate the effects of ethanol exposure on the foetus, it has been proposed that fortifying staple foods with choline might be a strategy to decrease the devastating effects of ethanol on the foetus [44]. This may be of benefit in a country such as South Africa that has very high levels of FASD. Hypertension was prevalent and the possible benefits of choline supplementation on hypertension management should be investigated. As it was found that participants who used ART were more likely to consume below adequate amounts of choline, use of ART, or HIV-infection, might be considered as a risk factor for a low choline intake. The median GBMI indicated that obesity is prevalent in the sample. Strategies to promote attaining a normal weight before conception require urgent attention, considering the various health risks associated with being overweight. Finally, pregnant women should be screened for psychological and social stress, and appropriate referrals and support provided in order to prevent short- and long-term adverse effects of maternal stress on their offspring.

## Data Availability

The datasets used and/or analysed during the current study are available from the corresponding author on reasonable request.

## Abbreviations

NuEMI study: Nutritional status of Expectant Mothers and their newborn Infants study
AI: adequate intake
ART: anti-retroviral medications
NCD: non-communicable disease
WHO: World Health Organization
SADHS: South African Health and Demographic Survey
HIV: human immunodeficiency virus
ANC: antenatal care
QFFQ: quantitative food frequency questionnaire
SA-FCDB: South African Food Composition Database
FAO: food and agriculture organization
BTT: Birth to Twenty study:GBMI:gestational body mass index
SD: standard deviations
IQR: interquartile range
CI: confidence intervals
EAR: estimated average requirement
IOM: Institute of Medicine
NAM: National Academy of Medicine
FASD: foetal alcohol spectrum disorder
CVD: cardiovascular disease
TMAO: trimethylamine N-oxide
